# The functions of autophagy at the tumour‐immune interface

**DOI:** 10.1111/jcmm.16331

**Published:** 2021-02-18

**Authors:** Xiaobo Luo, Yan Qiu, Palani Dinesh, Wang Gong, Lu Jiang, Xiaodong Feng, Jing Li, Yuchen Jiang, Yu L. Lei, Qianming Chen

**Affiliations:** ^1^ State Key Laboratory of Oral Diseases National Clinical Research Center for Oral Diseases Chinese Academy of Medical Sciences Research Unit of Oral Carcinogenesis and Management West China Hospital of Stomatology Sichuan University Chengdu China; ^2^ Department of Pathology West China Hospital Sichuan University Chengdu China; ^3^ Department of Periodontics and Oral Medicine Department of Otolaryngology–Head and Neck Surgery Rogel Cancer Center the University of Michigan Ann Arbor MI USA

**Keywords:** autophagy, immune cell, tumour cell, tumour immunity

## Abstract

Autophagy is frequently induced in the hypoxic tumour microenvironment. Accumulating evidence reveals important functions of autophagy at the tumour‐immune interface. Herein, we propose an update on the roles of autophagy in modulating tumour immunity. Autophagy promotes adaptive resistance of established tumours to the cytotoxic effects of natural killer cells (NKs), macrophages and effector T cells. Increased autophagic flux in tumours dampen their immunogenicity and inhibits the expansion of cytotoxic T lymphocytes (CTLs) by suppressing the activation of STING type I interferon signalling (IFN‐I) innate immune sensing pathway. Autophagy in suppressive tumour‐infiltrating immune subsets maintains their survival through metabolic remodelling. On the other hand, autophagy is involved in the antigen processing and presentation process, which is essential for anti‐tumour immune responses. Genetic deletion of autophagy induces spontaneous tumours in some models. Thus, the role of autophagy is context‐dependent. In summary, our review has revealed the dichotomous roles of autophagy in modulating tumour immunity. Broad targeting of autophagy may not yield maximal benefits. The characterization of specific genes regulating tumour immunogenicity and innovation in targeted delivery of autophagy inhibitors into certain tumours are among the most urgent tasks to sensitize cold cancers to immunotherapy.

## 1 BACKGROUND

**FIGURE 1:**
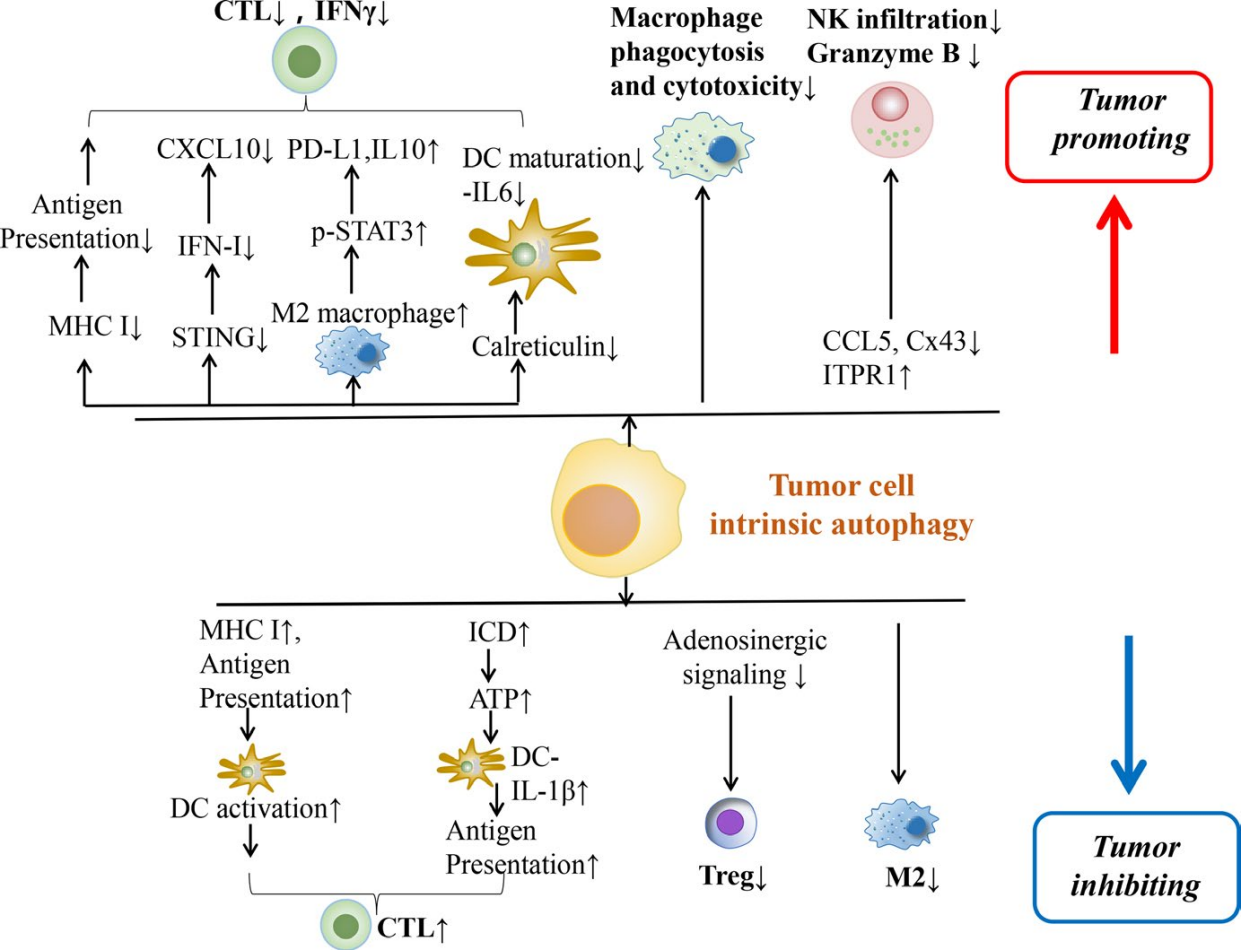
Schematic presentation regarding the potential mechanisms of tumour cell or immune cell instrinsic autophagy in modulating tumour‐immune interplay and the development of tumour. ATP, adenosine triphosphate; CCL5, chemokine (C‐C motif) ligand 5; CTL, cytotoxic T lymphocytes; Cx43, Connexin 43; DC, dendritic cell; ICD, immunogenic cell death; IFN‐I, type I interferon; ITPR1, inositol 1,4,5‐trisphosphate receptor, type 1; MHC, major histocompatibility class; NK, natural killer cells; PD‐L1, programmed death‐ligand 1; STAT3, signal transducer and activator of transcription 3; Treg, regulatory T cells

**FIGURE 2:**
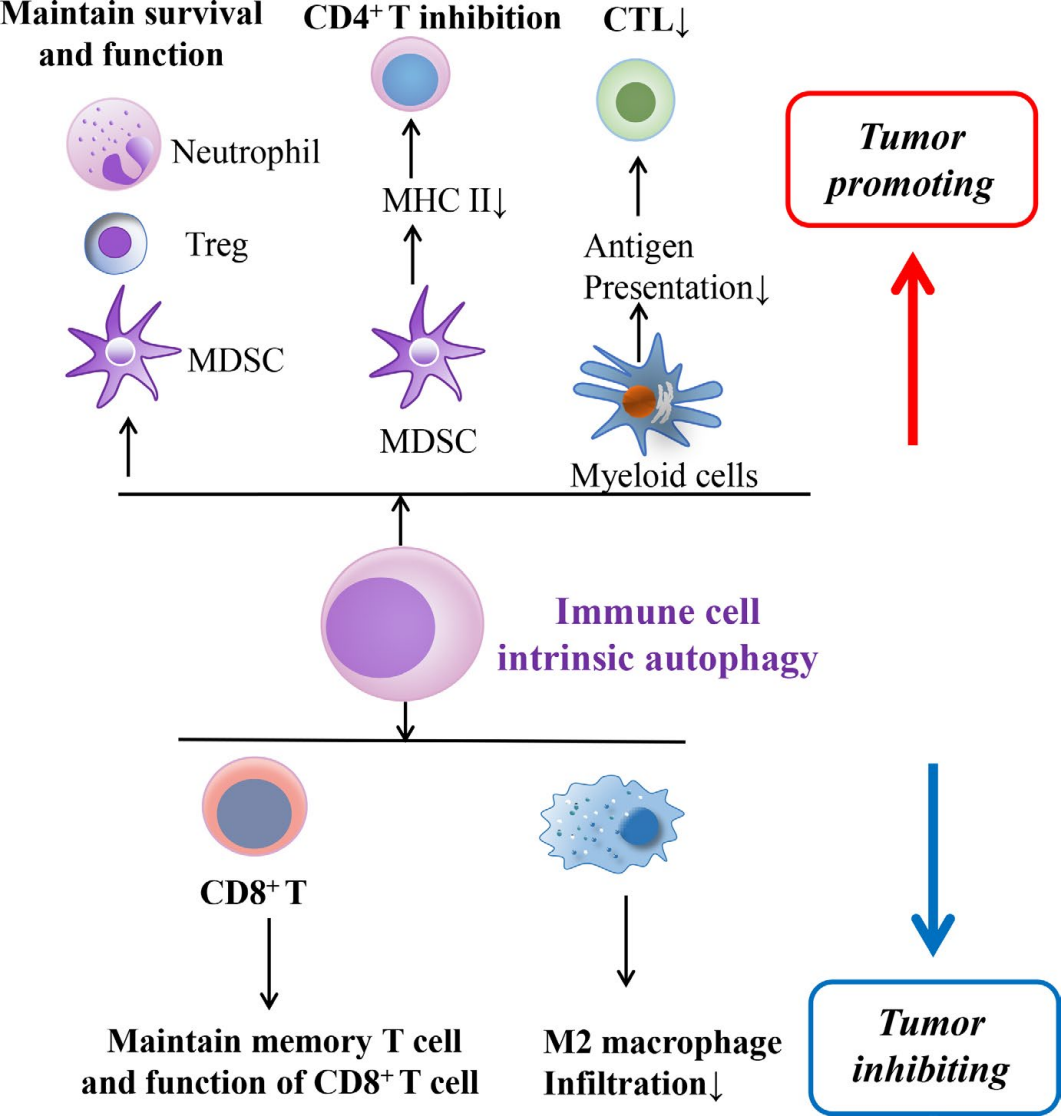
Schematic diagram indicating the possible mechanisms of immune cell instrinsic autophagy in regulating tumour‐immune interplay and the tumour outcome. CTL, cytotoxic T lymphocytes; MDSC, myeloid‐derived suppressor cells; MHC, major histocompatibility class; Treg, regulatory T cells

**FIGURE 3:**
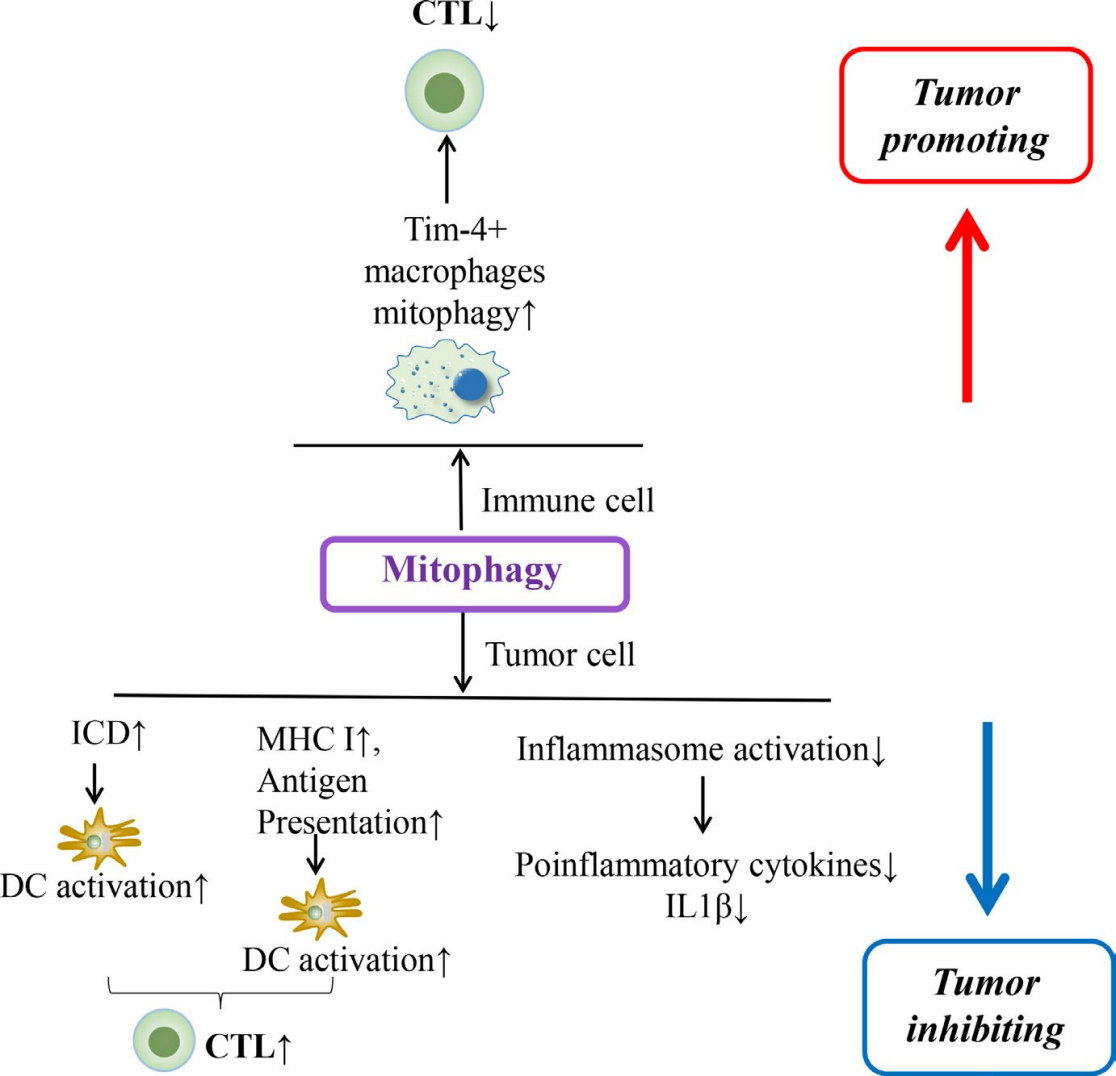
Schematic image demonstrating the potential role of mitophagy in regulating tumour immunity and the tumour outcome. CTL, cytotoxic T lymphocytes; DC, dendritic cell; ICD, immunogenic cell death; MHC, major histocompatibility class

Autophagy serves as an evolutionarily conserved physiological phenomenon to maintain cellular homeostasis and survival during nutrient deprivation. The initiation of autophagic response is briefly presented as the encapsulation of excessive or damaged cellular components and organelles into autophagosome leading to enzymatic degradation.^1^, ^2^ According to the various delivering routes and contents to lysosomes, autophagy is generally categorized into macroautophagy (predominant form generally termed as autophagy), microautophagy and chaperon‐mediated autophagy.^3^ Autophagy is also frequently altered under pathological circumstances, namely hypoxia, endoplasmic reticulum (ER) stress, nutrient deficiency, radiation and chemotherapy.^4^, ^5^, ^6^ Aside from its direct effect on cancer cell response to environmental challenges, recent studies show that autophagy in cancer cells regulates tumour‐immune interactions, depending upon the context of cancer types, metabolic alterations in the tumour microenvironment (TME) and the stage of cancers.^7^ Genetic evidence showed that autophagy is a critical mechanism suppressing tumour initiation,^8^ however, in established tumours autophagy contributes to adaptive resistance.^9^ Of note, mitophagy, another form of autophagy, plays a similar role in regulating tumour development by adjusting tumour immune response.^10^ In this review, we seek to summarize recent evidence characterizing the functions of autophagy, including mitophagy, in regulating tumour‐immune interactions (Figures 1, 2, 3; Table 1).

**TABLE 1:** Summary of literatures regarding tumour or immune cell‐intrinsic autophagy in the modulation of tumour immunity

Mechanisms involved in regulating tumour immunity	Tumour type	Source of autophagy	Authors	Year	The impact of autophagy on tumours
HPV16 E7‐STING and IFN‐I↓‐CTLs suppression MHC‐I degradation‐antigen presentation↓‐ CTL↓ Mitophagy‐CTL inhibition Mitophagy‐ICD‐ CTL and DC activation↑	HNSCC Pancreatic cancer Ovarian cancer Hepatocellular carcinoma	HNSCC Pancreatic cancer Tim‐4+ macrophages Hepatocellular carcinoma	Luo et al^31^ Yamamoto et al^42^ Xia et al^10^ Yu et al^74^	2020 2020 2020 2020	Tumour‐promoting Tumour‐promoting Tumour‐promoting Tumour‐inhibiting
p‐STAT3↑‐PD‐L1↑‐CTL inhibition MHC‐I and antigen presentation↑‐DC activation Mitophagy‐inflammasome activation↓	Lung adenocarcinoma Glioblastoma Hepatocellular carcinoma	Lung adenocarcinoma Glioblastoma Hepatocellular carcinoma	Liu et al^46^ Li et al^67^ Li et al^73^	2019 2019 2019	Tumour‐promoting Tumour‐inhibiting Tumour‐inhibiting
M2 macrophage‐TLR4‐mediated MyD88↑‐p38↑‐STAT3↑‐PD‐L1, IL10↑‐CTL↓ SOX2‐STING↓‐IFN‐I↓‐CD8^+^ T cells↓ Macrophage phagocytosis and cytotoxicity↓ Macrophage phagocytosis and cytotoxicity↓ and CD8^+^ T cells↓ Maintain function of MDSC and MHC‐II↓,CD4 T cells inhibition Mitophagy‐MHC‐I and antigen presentation by DCs↑	Melanoma HNSCC Glioblastoma Glioblastoma Melanoma Colorectal cancer	TRAPs HNSCC Glioblastoma Glioblastoma MDSC Colorectal cancer	Wen et al^24^ Tan et al^30^ Zhang et al^21^ Zhang et al^23^ Alissafi et al^58^ Ziegler et al^68^	2018 2018 2018 2018 2018 2018	Tumour‐promoting Tumour‐promoting Tumour‐promoting Tumour‐promoting Tumour‐promoting Tumour‐inhibiting
Macrophage phagocytosis and cytotoxicity↓ Tumour‐derived CCL5↓‐NK cell infiltration↓ Maintaining memory T cell‐CD8^+^ T cells↑ upon stimulation	NSCLC Melanoma Breast cancer	NSCLC Melanoma CD8^+^ T cells	Zhang et al^22^ Mgrditchian et al^18^ Curry et al^71^	2017 2017 2017	Tumour‐promoting Tumour‐promoting Tumour‐inhibiting
Inhibiting mTORC1 and c‐Myc function and glycolytic metabolism‐maintaining Treg function Maintain survival and function of MDSCs	Colon adenocarcinoma Breast cancer	Treg MDSC	Wei et al^56^ Parker et al^57^	2016 2016	Tumour‐promoting Tumour‐promoting
Cx43 in tumour cells↓‐NK cells↓ Maintain survival and function of neutrophils	Melanoma Hepatocellular carcinoma	Melanoma Neutrophils	Tittarelli et al^15^ Li et al^60^	2015 2015	Tumour‐promoting Tumour‐promoting
Autophagy sensor ITPR1↑‐NK‐derived granzyme B degradation Adenosinergic signalling↓‐Treg↓ in early phase of tumorigenesis	Renal cancer NSCLC	Renal cancer NSCLC	Messai et al^17^ Rao et all^61^	2014 2014	Tumour‐promoting Tumour‐inhibiting
Granzyme B released into tumour cells by NK cells↓ CTL cytotoxicity↓ Calreticulin ↓‐maturation of IL‐6 secreting DCs, IFNγ released by CTLs↓ Antigen presentation of myeloid cells↓‐CTL↓ Infiltrated M2 macrophage↓	Breast cancer Breast cancer Bladder cancer, cervical cancer and melanoma Colon cancer, melanoma Hepatocellular carcinoma	Breast cancer Breast cancer Tumour cells Myeloid cells Macrophage	Baginska et al^12^ Akalay et al^47^ Garg et al^50^ Baghdadi et al^59^ Lin et al^70^	2013 2013 2013 2013 2013	Tumour‐promoting Tumour‐promoting Tumour‐promoting Tumour‐promoting Tumour‐inhibiting
Antigen presentation by DCs↑‐CD8^+^ T cells↑	Breast cancer and lung cancer	TRAPs	Li et al^65^	2012	Tumour‐inhibiting
p‐STAT3↑‐tumour susceptibility to CTL‐mediated lysis↑ IFN‐I↓‐CD8^+^ T cells↓, CXCL10↓ ICD↑‐ATP release into TME↑‐IL‐1β from DCs↑‐Tumour lysis‐DC phagocytosis and antigen presentation↑‐CTL activation↑	Lung cancer and melanoma Breast cancer Colon cancer	Lung cancer and melanoma Breast cancer Colon cancer	Noman et al^45^ Wei et al ^41^ Michaud et al^53^	2011 2011 2011	Tumour‐promoting Tumour‐promoting Tumour‐inhibiting
p‐STAT3↑‐DC maturation↓‐CTL cytotoxicity↓	Melanoma and lung cancer	Melanoma and lung cancer	Yu et al^43^	2007	Tumour‐promoting

Note

Abbreviations: ATP, adenosine triphosphate; CCL5, chemokine (C‐C motif) ligand 5; CTL, cytotoxic T lymphocytes; Cx43, Connexin 43; DC, dendritic cell; HNSCC, head and neck squamous cell carcinoma; ICD, immunogenic cell death; IFN‐I, type I interferon; ITPR1, inositol 1,4,5‐trisphosphate receptor, type 1; MDSC, myeloid‐derived suppressor cells; MHC, major histocompatibility class; mTORC1, mammalian target of rapamycin complex 1; NK, natural killer cells; NSCLC, non–small cell lung cancer; PD‐L1, programmed death‐ligand 1; STAT3, signal transducer and activator of transcription 3; TLR4, Toll‐like receptor 4; TME, tumour microenvironment; TRAPs, tumour cell‐released autophagosomes; Treg, regulatory T cells.

## 2 AUTOPHAGY IN ESTABLISHED TUMOURS PROMOTES EVASION FROM INNATE AND ADAPTIVE IMMUNE SURVEILLANCE

Autophagy could directly or indirectly exert its effect on the innate immunity mediated by natural killer (NK) cells, dendritic cells (DCs) and macrophage population. First, autophagy of tumour cells promotes adaptive resistance to NK‐induced tumour lysis. NK cells, which are considered as the first‐line defence against tumours, releasing perforin, and granzyme B for the lysis of tumour cells.^11^, ^12^ Its anti‐cancer role has been validated in malignancies, such as gastric cancer^13^ and lung cancer.^14^ By exploiting the in vivo and in vitro breast cancer models, Baginska et al observed that the autophagy provoked by the hypoxic TME is involved in the degradation of granzyme B originated from NK into cancer cells, thus counteracting the apoptotic cell death effect induced by NK cells.^12^ Besides, several studies also suggest additional mechanisms that contribute to the low tumour immunosurveillance and cytotoxicity of NK cells for cancers. Gap junctions (GJs) are interacting channels that mediate the exchange of the small molecules between cells composed by connexin subunits, among which Connexin 43 (Cx43) is uncovered as the major GJ protein located at the immunological synapse and bridging the interplay between immune cells and cancer cells.^15^ Hypoxia‐induced autophagic flux results in the degradation of Cx43 in melanoma cells and impairs the cytotoxic effects of NK cells upon cancer cells. In agreement, elevated Cx43 expression levels in tumour cells are beneficial to enhance the efficacy of NK‐based immunotherapy.^15^ Inositol 1,4,5‐trisphosphate receptor, type 1 (ITPR1), as one ligand‐gated channel of ion for managing calcium release from the endoplasmic reticulum, is reported to be able to induce autophagy.^16^ Messai's study regarding clear cell renal cell carcinomas (CCRCC) indicated that the elevated expression of ITPR1 evoked by HIF‐2α initiated the autophagic degradation of granzyme B and abolished the NK‐induced killing effect on tumour cells. In agreement with that, they implanted the tumours in mice and observed reduced tumour growth by inhibiting ITPR, while the depletion of NK cells reverted the tumour suppression.^17^ As another line of evidence of autophagy‐mediated immune resistance, depletion of autophagy‐promoting Beclin 1 (BECN1) leads to increased intensity of chemokine (C‐C motif) ligand 5 (CCL5) expression within melanoma cells and redirects massive NK cells into the tumour microenvironment, thus leading to tumour suppression.^18^

Macrophages may also exert innate immune surveillance in the TME through their phagocytic functions.^19^, ^20^, ^21^ A glioblastoma study employing a combinatorial treatment to target both VEGF and CD47, the latter of which inhibited the phagocytic effect of macrophages, revealed that it could trigger autophagy of cancer cells which attenuated the phagocytosis and cytotoxicity of macrophage population. Inhibition of various signalling pathways, including Akt/mTOR and Erk, was responsible for the enhanced autophagy.^21^ The same group also demonstrated that the combination of anti‐CD47 therapy with autophagy inhibitor would robustly improve the therapeutic efficacy against non–small cell lung cancer (NSCLC).These results suggest that autophagy originated from tumour cells could impede the phagocytic function of macrophages.^22^ Zhang et al found that autophagy occurring in glioblastoma cells could mitigate the immunotherapeutic efficacy of anti‐CD47‐SIRPα treatment, displaying as the reduced macrophage‐derived phagocytosis and subsequent attenuation of CD8^+^ T‐cell cytotoxicity.^23^ Notably, macrophages may be regulated by tumour cell‐released autophagosomes (TRAPs) and affect the cytotoxic T lymphocytes (CTLs).^24^ TRAPs are a type of double‐membrane vesicles released into the TME by tumour cells, which escape from the lysosome fusion stage of classical autophagy.^25^ Wen and colleagues uncovered that within several tumour models, the TRAPs could skew macrophages into M2‐phenotype with higher levels of PD‐L1 and IL‐10 via Toll‐like receptor 4 (TLR4)‐MyD88‐p38‐STAT3 pathway, therefore resulting in suppression of CTL function and reduced IFN‐γ secretion.^24^

Moreover, tumour‐associated autophagy also contributes to evasion from adaptive immunity. For example, the response rate of head and neck squamous cell carcinoma (HNSCC) to immunotherapy remains less than 15%, for which low immunogenicity and a poor infiltration of CTLs were indicated as the possible reason.^26^, ^27^ Type I interferon (IFN‐I) signalling promotes anti‐tumour effects by mediating the recruitment and maturation of antigen‐presenting cells (APCs). Stimulator of IFN genes (STING) is a pivotal adaptor protein that could activate the IFN‐I pathway.^28^, ^29^ Nonetheless, STING is frequently inhibited in TME, contributing to tumour escape from innate immune sensing. Recent studies identified previously unknown functions of oncogenes in suppressing the STING‐IFN‐I innate immune sensing pathway.^30^, ^31^ Specifically, SOX2, previously known as a cancer stemness gene, was correlated with immunosuppression.^30^, ^32^, ^33^ *SOX2* amplification in tumour cells leads to an increased autophagic influx, which promoted the turnover of STING in HNSCC cells. Inhibition of autophagy could rescue SOX2‐potentiated suppression of STING. In addition, the results of in vivo experiment suggested that SOX2‐expressing tumours contained lower numbers of CD8^+^ CTLs and that those infiltrating T cells expressed higher levels of PD‐1 than SOX2‐negative tumours.^30^ HPV^+^ HNSCC is driven by a distinct aetiology, with different immune infiltration patterns from HPV^‐^ tumours. Interestingly, HPV^+^ HNSCCs contain less T‐cell receptor richness, in contrast to its usually heavy immune infiltration.^34^ IFN‐I is essential for tumour‐specific CTL expansion. A recent study showed that HPV16 E7 could contribute to the autophagic degradation of STING by binding to NLRX1, which was shown to promote autophagosome formation.^5^, ^31^, ^35^, ^36^ NLRX1 deficiency in the tumour cells promoted CD8^+^ CTL expansion located in the tumour‐draining lymph nodes and reduces CTL exhaustion in the TME.^31^ In agreement, additional studies also found that high‐risk HPV subtypes utilize a number of strategies to antagonize IFN‐I induction^37^, ^38^, ^39^; Gariglio and colleagues observed that HPV E7 could attenuate the IFN‐I activation in HPV‐transformed cells via epigenetic silencing of sensor genes including RIG‐I, cGAS and STING in an SUV39H1‐dependent manner.^37^ NLRX1 has an LC3‐interacting region and can directly interact with LC3. Such interaction underpins an NLRX1‐mediated mitophagy process. Depletion of NLRX1 promotes mitochondria‐derived reactive oxygen species, which arguably amplifies the production of Th1 cytokines.^40^ The role of autophagy in inhibiting IFN‐I was also corroborated using a transgenic FIP200 (FAK family‐interacting protein of 200 kD)‐deficient mouse model. *FIP200* is an essential autophagy gene, in the absence of which mammary tumorigenesis is suppressed. The study found that inhibition of autophagy promoted the activation of IFN‐I signalling as well as its downstream chemokines such as CXCL10, subsequently inducing CD8^+^ CTL expansion in the TME.^41^ Of interest, Yamamoto and colleagues recently reported that autophagy is responsible for the degradation of MHC‐I in pancreatic ductal adenocarcinoma by employing the autophagy cargo receptor NBR1, resulting in the tumour immune evasion.^42^ Thus, modulating selective autophagy represents a non‐tapped approach to fine‐tune host immune responses.

Additional evidence implies that autophagy is also responsible for tumour immune escape by stimulating signal transducer and activator of transcription 3 (STAT3) signalling, an oncogenic pathway. The STAT3 pathway has been an important link between tumour and immune cells.^7^, ^43^ Wang et al reported that STAT3 activation occurring in tumour cells could significantly reduce the production of pro‐inflammatory cytokines and chemokines critical for APC maturation and its recruitment to the tumour bed.^44^ Autophagy has been shown to increase STAT3 phosphorylation in multiple tumour models.^45^, ^46^, ^47^ Autophagy may also inhibit adaptive immunity by dampening the immunogenic cell death (ICD)‐induced immune killing. ICD can be triggered by several anti‐cancer treatments such as chemotherapy, radiotherapy^48^, ^49^ and hypericin‐based photodynamic therapy (Hyp‐PDT).^50^ This phenomenon is predominantly represented as the calreticulin (CRT) exposure on the cellular surface, the secretion of high mobility group box 1 (HMGB1) along with adenosine triphosphate (ATP). ^51^, ^52^, ^53^ These are pivotal to the proper processing of antigen by APCs, and these molecules, including CRT, HMGB1 and ATP were defined as damage‐associated molecular patterns (DAMPs).^54^ Garg et al reported that by genetically blocking autophagy in the tumour model under Hyp‐PDT, an increase in CRT and ICD‐caused immune reaction was detected. This was elucidated as the up‐regulation of IL6‐producing mature DCs and CTLs along with IFN‐γ.^50^

In addition to tumour‐intrinsic autophagy, immune cell–inherent autophagy may also deliver resistance to immune killing. Myeloid‐derived suppressor cells (MDSCs) and regulatory T cells (Tregs) are the dominant subsets in the TME to promote tumour immune escape.^55^, ^56^ HMGB1‐induced autophagy was found essential for maintaining the survival of MDSCs in the TME.^57^ Autophagy could also induce the lysosomal breakdown of MHC‐II and repress the anti‐tumour effect of CD4^+^ T cells.^58^ As a critical adaptive mechanism in a nutrient‐poor environment, autophagy in the MDSCs and Tregs is essential to maintain their survival and sustained production of transforming growth factor‐β (TGF‐β), which dampens the activation of CTLs.^56^, ^59^, ^60^

## 3 THE PROTECTIVE ROLE OF AUTOPHAGY IN PROMOTING NEOANTIGEN PRESENTATION

Compelling evidence demonstrates that the functions of autophagy in tumour initiation and established tumour response to therapy are different. One of the examples is that the genetic deletion of *BECN1* enhances spontaneous tumour formation.^61^ A recent study suggests that such autophagy‐mediated protection depends on immune surveillance. Autophagy promotes the processing and presentation of neoantigens from transforming cells to CTLs, leading to the elimination of target cells.^62^ Under the circumstances of compromised proteasomal function, autophagy is central for the assembly of neoantigens with MHC‐I complex in APCs to facilitate its cross‐presentation to CD8^+^ T cells.^4^, ^63^, ^64^ In addition, autophagy in transforming cells facilitates antigen presentation to CD8^+^ T cells.^65^, ^66^ Here, we summarize some evidence for the above notion and other potential mechanisms of autophagy that contribute to anti‐tumour immunity.

The efficient uptake and presentation of tumour antigen is essential to subvert the immunosuppressant TME. Li and colleagues showed that tumour cell autophagy triggered by the synthetic Nano‐DOX contributed to the increased immunogenicity of glioblastoma. These are presented as the elevated expression of MHC‐I complex and antigen presentation on tumour cells, the activation of DCs, and the transmission of DAMPs into extracellular TME.^67^ Michaud et al observed that autophagy of colon cancer cells could promote ICD, including the ATP release followed by IL1‐β released from activated DCs, and the latter cytokine might enhance DC functions.^53^ Additionally, the autophagosome extruded by tumour cells, called TRAPs could also implicate in this process. One study indicated that TRAPs produced by alpha‐tocopheryloxyacetic acid (a‐TEA) treatment in breast and lung cancer models might boost the potential of DCs to intake and present antigens, then inducing the activation of CD8^+^ T cells.^65^ Autophagy‐mediated reduction of lysosomal integrity could potentiate MHC‐I presentation and augment the cross‐dressing of MHC‐antigen complexes to DCs, contributing to significant CD8^+^ T‐cell activation.^68^ To address the tumour stage–dependent dichotomous roles of autophagy, genetically engineered mouse models offer a robust tool. For example, in the early stage of carcinogenesis of *KRas^G12D^* murine lung cancer, autophagy inhibited Treg infiltration through suppressing adenosinergic signalling and repressed tumour growth.^61^ However, the autophagy at later stage potentiated tumour progression via dampening oxidative stress as well as inhibiting the DNA damage response.^61^

Similar to the observation in tumour cells, autophagy in macrophages was shown to promote the surface expression of MHC‐II.^69^ In a diethylnitrosamine‐induced hepatocellular carcinoma model, autophagy in macrophages was essential for their intratumoral infiltration.^70^ Another study reported that autophagy of T cells induced by metformin in a breast cancer model of mice could substantially enhance the functional CD8^+^ T‐cell response by maintaining T‐cell function; meanwhile, the autophagy of CD8^+^ memory T cells is considered indispensable to maintain their survival and sustain tumour immunosurveillance after tumour resection.^71^

## 4 THE CRUCIAL ROLE OF MITOPHAGY IN REGULATING TUMOUR IMMUNE RESPONSE

Autophagy‐mediated turnover of aged and/or damaged mitochondria is known as mitophagy.^72^, ^73^ The role of mitophagy in modulating the tumour immunity is emerging. On one side, Ziegler and colleagues show that mitophagy promotes anti‐tumour immunity. Increased mitophagy in intestinal epithelial cells triggers iron accumulation–induced lysosomal membrane permeabilization, which promotes the release of proteases into the cytosol and augments of MHC class I presentation.^68^ Besides, in the hepatocellular carcinoma (HCC) model, mitophagy could be induced upon the icaritin treatment, which subsequently triggers ICD and augments anti‐tumour immunity.^74^ On the other hand, mitophagy can also suppress inflammation. FUN14 domain‐containing 1 (FUNDC1), one mitophagy receptor that initiates the mitophagy, suppresses inflammasome activation and related immune responses.^73^ In addition, Xia and colleagues uncovered that in mice ovarian cancer models with peritoneal metastasis, the infiltrating Tim4+ tumour‐associated macrophages (TAMs) exhibited higher mitophagy activity, thereby inhibiting the T cell–mediated anti‐tumour immunity and facilitating tumour progression.^10^ Thus, mitophagy may regulate different inflammatory pathways where mitochondria maintains their homeostasis.^75^ Its role in tumour cells and immune cells likely impose different impacts on anti‐tumour immunity (Figure 3). Future different studies using genetically engineered models, syngeneic models and human material are needed to better refine the role of mitophagy of different cell types in regulating tumour immunogenecity.

## 5 UPSTREAM REGULATORS OF AUTOPHAGY INVOLVED IN THE TUMOUR IMMUNE RESPONSE

In TME, autophagy can be induced by several stress factors, including hypoxia, endoplasmic reticulum (ER) stress, nutrient deprivation, extracellular matrix (ECM) disassociation and DAMPs.^57^, ^76^, ^77^, ^78^, ^79^ Hypoxia is revealed in approximately 50%‐60% tumours, and several hypoxia‐mediated pathways are reported to induce autophagy.^80^, ^81^ HIF1α translocates into nucleus under hypoxic conditions, resulting in increased adenovirus E1B 19 kD‐interacting protein 3 (BNIP3) and its interacting partner BNIP3L. The BNIP3‐BNIP3L complex promotes autophagy in a BECN1‐dependent fashion.^82^ In relation to that, another study found that NANOG could transcriptionally improve the level of BNIP3L, thereby inducing autophagy and abolishing the CTL‐mediated tumour lysis.^80^ With the increased ratio of ADP:ATP within the hypoxic TME, adenosine monophosphate–activated protein kinase (AMPK) could be activated to stimulate autophagy via attenuation of the mammalian target of rapamycin (mTOR) pathway.^83^, ^84^

Another process closely associated with hypoxia, epithelial to mesenchymal transition (EMT) is another inducer of autophagy in TME, which confers tumour resistance to CTL killing. EMT of cancer cells accompanied with Snail homolog 1 (SNAI1) overexpression up‐regulates BECN1, leading to increased autophagy.^85^, ^86^ EMT could activate autophagy through regulating genes of DAPK1, PTEN and CDKN2A, enabling the cancer evasion from CTL cytotoxicity.^47^

HMGB1, as an inducer of ICD, can trigger autophagy in TME. A co‐culture study revealed that HMGB1 could induce autophagy in colon cancer cells in an ER stress‐JNK phosphorylation‐dependent manner.^78^ Another study implied that HMGB1, similar to BNIP3, dissociated Bcl2 from BECN1, which in turn triggered autophagy.^79^

Mitophagy in tumours may be modulated by other upstream modulators. For instance, the STAT3 status, the FUNDC1 expression and the icaritin treatment implicate in regulating mitophagy and tumour immunity.^68^, ^73^, ^74^ In addition, high expression levels of arginase‐1 suppress mTORC1 activation, which then contributes to enhanced mitophagy level in TAMs.^10^

## 6 CONCLUSIONS

In summary, despite the dichotomous functions of autophagy in regulating anti‐tumour immune responses, its predominant function is likely dependent on cancer stages, cancer types, immune infiltration profiles and modelling methods. Autophagy in immune cells is an essential protective mechanism by facilitating tumour neoantigen presentation. However, autophagy in cancer cells may promote adaptive resistance to immune killing by dampening IFN‐I‐mediated immune sensing and rapid turnover of cytotoxic effector molecules. Global inhibition of autophagy may not yield the maximal benefits due to its interference with the antigen presentation machinery in the APCs; even such inhibition may sensitize tumours to immune killing. Thus, the characterization of specific genes regulating tumour immunogenicity and innovation in targeted delivery of autophagy inhibitors into tumour cells are among the most urgent tasks to sensitize cold cancers to immunotherapy.

## CONFLICT OF INTEREST

The authors declare no potential conflicts of interest.

## AUTHOR CONTRIBUTIONS

**Xiaobo Luo:** Conceptualization (lead); Funding acquisition (lead); Writing‐original draft (lead); Writing‐review & editing (lead). **Yan Qiu:** Conceptualization (equal); Writing‐original draft (lead); Writing‐review & editing (equal). **Palani Dinesh:** Writing‐original draft (equal); Writing‐review & editing (equal). **Wang Gong:** Writing‐original draft (equal). **Lu Jiang:** Writing‐review & editing (equal). **Xiaodong Feng:** Writing‐review & editing (equal). **Jing Li:** Writing‐review & editing (equal). **Yuchen Jiang:** Writing‐review & editing (supporting). **Yu L. Lei:** Conceptualization (equal); Funding acquisition (equal); Supervision (lead); Writing‐review & editing (lead). **Qianming Chen:** Conceptualization (equal); Funding acquisition (equal); Supervision (lead); Writing‐review & editing (lead).
